# Electric wire as a urethral foreign body: A case report

**DOI:** 10.1097/MD.0000000000028103

**Published:** 2021-12-03

**Authors:** Qiqi Song, Jingwen Zhang, Ronghong Jiao

**Affiliations:** a Hebei Medical University, Shijiazhuang, China; b Department of Ultrasound, Hebei General Hospital, Shijiazhuang, China.

**Keywords:** foreign body, ultrasound, urethra

## Abstract

**Rationale:**

Most self-insertion of urethral foreign bodies is the result of exotic impulses, psychometric problems, sexual curiosity, or sexual practice while intoxicated. Ultrasound has been proven to be an effective tool for determining the presence, location, and characteristics of the urethral foreign body.

**Patient concerns:**

A 48-year-old man presented with a mass in the urethra for 2 years. Physical examination suggested swelling and ulcer in the scrotum. The white blood cell count was elevated (12.60 × 10^9^/L). Urinalysis showed an increased white cell count (484.60/μL) and urine occult blood (±).

**Diagnosis:**

Ultrasound examination of the genitourinary system identified an 8.3 cm linear hyperechoic object and hyperechoic spots in the urethra. Computed tomography revealed an extremely hyperdense lesion in the penis. Intraoperative findings showed electric wire bending and winding surrounded by fibrous tissues with urethral rupture.

**Interventions:**

The patient was treated with urethrotomy as the endoscopic treatment failed. and the electric wire was removed successfully.

**Outcomes:**

The patient was followed up for 45 days without discomfort. Ultrasound examination of the genitourinary system suggested that the wound was almost healed, but with a small urethral effusion, with a maximum depth of approximately 1.9 mm.

**Lessons:**

A foreign body was inserted into the patient's urethra for 2 years without any medical treatment. Urethral perforation was found during surgery. In such cases, ultrasound examination can determine the location, shape, and size of the urethral foreign body and play an important role in the diagnosis of foreign bodies.

## 1 Introduction

A urethral foreign body is not a rare condition in the clinic, but often has many serious complications, such as urinary tract infection, acute urinary retention, or even urinary interruption. Bearelly et al^[1]^ studied the characteristics of genitourinary foreign body self-insertion and found that the peak ages were between 18 and 55 years old, and most patients had low income levels. The urethral foreign bodies reported in the literature vary. Loufopoulos et al^[2]^ reported a 15-year-old boy inserted a USB wire into the urethra to measure the length of his penis. Garg et al^[3]^ reported a 21-years old man with a urethral electric wire with dysuria and gross hematuria. In these cases, the foreign body was quickly diagnosed and removed. However, a case of a 48-year-man who inserted a foreign body into the urethra by himself for 2 years has been reported.

## 2 Case report

### 2.1 Patient information

This study was approved by the Human Ethics Committee of Hebei General Hospital, and informed written consent was obtained from the patient for publication of this case report.

A 48-year-old man was admitted with complaints of a mass in the right scrotum. The mass was discovered unintentionally 2 years ago, ruptured with white substance outflow a year ago, and he had no symptoms such as pain, dysuria, or urinary incontinence, and treatment was not administered. He denied a history of genetic disease or infection. After sonographic examination suggested a foreign body, medical history was asked again, and the patient was admitted that a cellphone charging cable was inserted into the urethra himself 2-years ago.

### 2.2 Clinical findings

Physical examination suggested swelling and ulcer in the scrotum. An irregular, firm mass was palpated in the scrotum and wrapped around the penis, with no fluctuation or activity. Blood tests revealed an elevated white blood cell count (12.60 × 10^9^/L), blood platelet count (363 × 10^9^/L), and neutrophil count (0.85 × 10^9^/L). Urinalysis showed an increased white cell count (484.60/μL) and urine occult blood (±).

### 2.3 Diagnostic assessment

**Figure 1:**
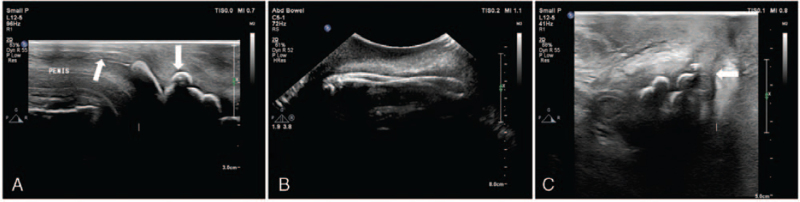
Ultrasonography features before surgical treatment. A. The high-frequency probe shows the posterior urethra and the arc strong echoic spot with shadowing in the middle urethra (arrows). B. Low-frequency probe shows the full view of the foreign body. C. The high-frequency probe revealed the damage of the central urethra (arrow) and urine leakage into the scrotal wall.

The patient was subjected to ultrasound of the genitourinary system. The examination was performed by an experienced ultrasound doctor. A Philips 7C Color Doppler ultrasound diagnostic instrument was used with a high-frequency probe, intermediate-frequency probe, and low-frequency probe. Ultrasound examination identified enhanced echoes from the middle urethra to the posterior urethra and several hyperechoic spots in the middle urethra. The linear hyperechoic object had an irregular shape. The low-frequency probe identified a linear hyperechoic object with a wide acoustic shadow in the middle urethra. The linear hyperechoic object was continuous with hyperechoic spots. The hyperechoic object at the posterior urethra was crooked as a “blind end” (Fig. 1A, 1 B). The size of the foreign body was approximately 8.3 cm. The hyperechoic body penetrated the middle cavernous body of the urethra and was partially located in the scrotum (Fig. 1C). The scrotum was swollen with an echo-free structure (1.1 × 0.7 cm). Ultrasonography revealed urethral foreign bodies with urethral injury and scrotal wall thickening associated with urine infiltration.

**Figure 2:**
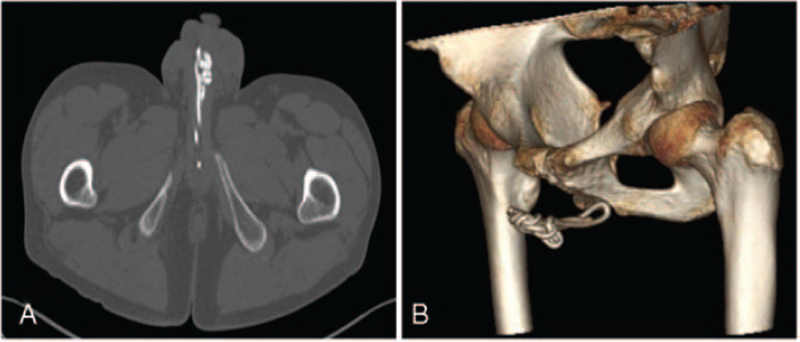
CT imaging of the sacrum. A. Axial image of CT of male genital organ. B. Volume rendering of CT.

A pelvic computed tomography (CT) scan showed an extremely hyper-dense lesion in the penis, with a CT numerical value of 1200HU, a patchy low-density shadow in the right scrotum (Fig. 2A, B). CT emphasized that urethral foreign bodies could be considered.

### 2.4 Treatment and follow-up

**Figure 3:**
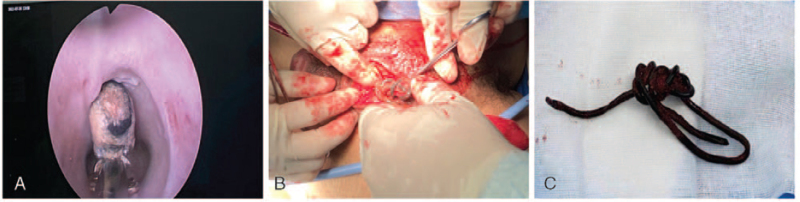
Intraoperative photograph. A. Endoscope showed the object. B. Surgical findings. Intraoperative findings showed the foreign body was bending and winding, and surrounded by fibrous tissues, urethral rupture. C. Tortuous coiled cellphone charging cable.

**Figure 4:**
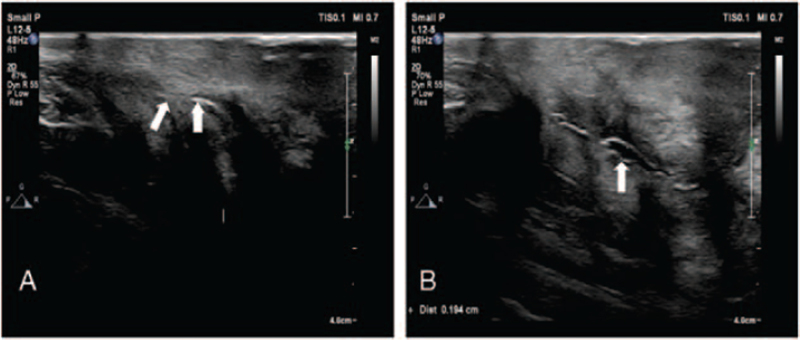
Postoperative ultrasonography at 45 days follow-up. A. The high frequency probe shows almost healed wound (arrows). B. The visualization of urethra in ultrasound scan suggested the presence of a small effusion(arrow).

An attempt was made to insert a cystoscope or a ureteroscope to remove the wire under general anesthesia but failed due to a shortage of space. The cystoscopy and ureteroscope showed a linear foreign body incarcerated in the urethra and was inserted into the scrotum (Fig. 3A). Then, a urethrotomy was performed, and the wire was removed (Fig. 3B, C). Intraoperative findings showed that the foreign body was bent, winding, and surrounded by fibrous tissues. An 18F catheter was placed in the bladder with normal urine drainage. The incision was sutured in layers. At the end of the procedure, a 16F catheter was inserted into the suprapubic fistula and ended with an inflated balloon in the bladder. The patient received anti-infective treatment following the surgery. At the 45th-day follow-up, the ultrasound of the genitourinary system was reviewed, and it was found that the wound was almost healed (Fig. 4A). Ultrasound revealed a small urethral effusion, with a maximum depth of urethral effusion was 1.9 mm (Fig. 4B).

## 3 Discussion

The interposition of foreign bodies into the urethra is rare in clinical practice. These foreign bodies were probably self-inflicted, accidentally introduced, or iatrogenic.^[4]^ Although numerous cases of self-inflicted foreign bodies have been reported in men, it has also been reported in women and children.^[5]^

Generally, most self-insertion of urethral foreign bodies is caused by exotic impulses, psychometric problems, sexual curiosity, or sexual behavior while intoxicated.^[6]^ In males, the objects usually retain for a long period in the urethra, but in the female, the object may be voided or passed into the bladder because of the length of the passage.^[7]^ A wide variety of objects have been reported, such as an AAA battery, plastics, speaker wire, open safety pins, a marble, a cotton swab, nail scissors, steel dining forks, and silicon.^[8–11]^

The symptoms of foreign bodies in the urethra vary from person to person, including lower abdominal pain, dysuria, external genitalia pain with or without swelling, hematuria, urinary frequency, urinary retention, urethral lacerations, urinary tract and sexually transmitted infections, dyspareunia, and fever.^[8,10,12,13]^ Without prompt treatment, the urethral foreign body might lead to a rectal abscess, periurethral abscess, urethral fistula, urethral stenosis, calcification of foreign bodies, calculus formation, gangrene, or even squamous cell carcinoma.^[12,14]^

The optimal approach for removing the urethral object depends on the size, location, extent of urinary tract injury, and type of object applied to the urethra.^[15,16]^ Various treatment methods of removal have been described, including voiding, manual compression of the urethra, transurethral and/or percutaneous endoscopic techniques, urethrotomy, Fogarty catheterization, and injection of solvents.^[5,10,12,16,17]^ Most urethral foreign bodies, especially objects in the anterior urethra, can be removed by endoscopic resection. However, for posterior urethral foreign bodies or foreign bodies with a severe inflammatory response, invasive procedures might be required.^[5,15,16]^

Due to feelings of guilt and humiliation, patients with a urethral foreign body always postpones search for help and conceals the medical history, which leads to serious complications or even misdiagnosis. In this case, the patient inserted the urethral foreign body for 2 years without medical treatment, and he did not have a history of foreign body insertion at the first presentation, which led to a delay in diagnosis, severe urethral damage, and failure of endoscopic attempts. Therefore, early diagnosis and appropriate consultation are crucial to the patients. Medical history should be carefully assessed to make an accurate diagnosis.

Imaging tests, including X-ray, ultrasound, CT, and magnetic resonance imaging, were used to examine the objects in the urethra. Ultrasound is a dynamic, real-time, noninvasive inspection technology that has been proven to be an effective tool to determine the size, location, and characteristics of urethral foreign bodies.^[18]^ At the same time, foreign bodies should be differentiated from urologic diseases such as urinary stones,^[19]^ which also revealed enhanced echoes with acoustic shadow, but they could cause sharp pain and hematuria. Moreover, urethral foreign bodies can occasionally lead to urinary stones. Noble et al.^[20]^ reported the case of a 73-year-old man with urethral calculus formed around the Foley catheter. It was reported that due to a foreign body covered with stones, two patients were diagnosed with urinary stone disease on radiologic imaging.^[21]^

For ultrasound examination, the proper selection of the probe is also crucial. In this case, three different frequency probes were used. A high-frequency probe was used to show the foreign body in the anterior urethra, and the intermediate-frequency probe was used to detect the foreign body and injuries of the middle and distal sections of the urethra, while the low-frequency probe was employed to present the full picture of the urethral object. Thus, a diagnosis of urethral foreign body and urethral injury was made. The comprehensive and correct diagnosis of this case fully demonstrates the superiority of the ultrasound technology.

## 4 Conclusion

A foreign body was inserted into the patient's urethra for 2 years without any medical treatment. Due to feelings of guilt and humiliation, the patient did not have a history of foreign body insertion at the first presentation, so appropriate consultations were crucial to the patients. Furthermore, in such cases, ultrasound examination could determine the location, shape, and size of the urethral foreign body and could be used as an important diagnostic technique.

## Author contributions

Ronghong Jiao designed the study, involved in the analysis and the interpretation of data, revised the draft manuscript, and revised the manuscript.

Qiqi Song wrote and revised the draft manuscript and subsequent manuscripts.

Jingwen Zhang assisted with drafting and revising the manuscript.

All authors read and approved the final manuscript.

Writing – original draft: Qiqi Song, Ronghong Jiao.

Writing – review & editing: Jingwen Zhang, Ronghong Jiao.
